# Combining dopamine and vitamin E enhances the differentiation to cholinergic neurons of mesenchymal stem cells

**DOI:** 10.1093/jnen/nlaf025

**Published:** 2025-04-11

**Authors:** Ramada R Khaswaneh, Ejlal Abu-El-Rub, Ayman Alzu’bi, Rawan Almazari, Amneh Alrabie, Fatimah A Almahasneh, Amani Kasasbeh, Heba F AI-jariri, Ayman Mustafa

**Affiliations:** Department of Basic Medical Sciences, Faculty of Medicine, Yarmouk University, Irbid, Jordan; Department of Basic Medical Sciences, Faculty of Medicine, Yarmouk University, Irbid, Jordan; Department of Basic Medical Sciences, Faculty of Medicine, Yarmouk University, Irbid, Jordan; Department of Basic Medical Sciences, Faculty of Medicine, Yarmouk University, Irbid, Jordan; Department of Basic Medical Sciences, Faculty of Medicine, Yarmouk University, Irbid, Jordan; Department of Basic Medical Sciences, Faculty of Medicine, Yarmouk University, Irbid, Jordan; Department of Basic Medical Sciences, Faculty of Medicine, Yarmouk University, Irbid, Jordan; Department of Basic Medical Sciences, Faculty of Medicine, Yarmouk University, Irbid, Jordan; Department of Basic Medical Sciences, College of Medicine, QU Health, Qatar University, Doha, Qatar

**Keywords:** cholinergic neurons, dopamine, mesenchymal stem cells, neuronal differentiation, vitamin E

## Abstract

Recent research indicates that mesenchymal stem cells (MSCs) can transdifferentiate into neuron-like cells under specific conditions, offering promise for neuronal regeneration. However, challenges remain in optimizing differentiation protocols to generate specific neuron types. This study explores the impact of supplementing neuronal induction media with dopamine and vitamin E to guide MSCs toward specific neuronal subtypes. Human adipose-MSCs were utilized to investigate neuronal differentiation. The cells were cultured in induction media supplemented with 2 concentrations of dopamine (2.5 and 5 µM) and vitamin E (12.5 and 25 µM). Immunostaining and western blot analysis were employed to assess the sequential expression of neuronal markers associated with various stages of maturation and development. These markers included Nestin, MAP2, NeuN, TBR1, SATB2, DAT, DBH, and CHAT. The results demonstrate, for the first time, that supplementing neuronal induction media with dopamine and vitamin E significantly enhances and accelerates the differentiation of MSCs into neuronal cells. Furthermore, the findings suggest that the induced cells are predominantly reprogrammed toward a cholinergic neuronal lineage. For MSCs, our study reveals that the addition of dopamine and vitamin E reprograms MSCs mainly toward cholinergic neurons, suggesting promising approaches for treating neurodegenerative disorders.

## INTRODUCTION

Treating neuronal disorders is profoundly challenging due to their complex nature and the lack of exact molecular mechanisms that can explain their pathogenesis. Neuronal diseases, such as Alzheimer disease, Parkinson disease, multiple sclerosis, and amyotrophic lateral sclerosis, affect the structure of neurons.^1^ As neurons are known for their poor ability to undergo regeneration, many neurological disorders are difficult to treat and become progressively worse, which negatively impacts the quality of life of affected patients.^2^

Inducing and conditioning mesenchymal stem cells (MSCs) to differentiate into functional neurons has gained significant attention in recent years driven by their potential therapeutic applications for neurodegenerative diseases and central nervous system injuries.^3^ MSCs are multipotent stem cells derived from various tissues, such as bone marrow, adipose tissue, and umbilical cord blood, and possess enormous abilities to differentiate into a variety of cell types, including bone, cartilage, and fat cells.^4^ As MSCs are not naturally predisposed to become neural cells, obtaining mature neurons or neuron-like cells from MSCs is challenging and requires investigations to explore promising growth factors and suitable culturing conditions that can promote their differentiation to neuronal cells.^3^

MSCs have been shown to express dopamine receptors on their surface, which play a pivotal role in regulating their functions and behavior.^5^ These receptors, including both D1-like and D2-like subtypes, are involved in key cellular processes such as proliferation, differentiation, and migration.^6^ The expression of dopamine receptors enables MSCs to respond to dopaminergic signaling, which in turn influence their interaction with the surrounding microenvironment.^7^ Dopamine, a neurotransmitter essential for neural signaling, can exert its effects primarily by binding to these receptors; it may impact the neuronal differentiation of MSCs through modulating neurogenesis signaling pathways.^8^ Furthermore, the role of dopamine in dictating the differentiation fate of MSCs toward neurons has not been thoroughly investigated.^9^

Vitamin E plays a role in promoting neural differentiation through activating important signaling pathways, transcription factors, and lipid metabolism that are crucial for neuronal development.^10^ Moreover, vitamin E enhances the ability of MSCs to develop the proper membrane composition required of functional neurons.^11^ It has been reported that vitamin E can activate the PI3K/Akt and MAPK/ERK pathways, which are essential for the activation of neuron-specific genes and proteins, including neurofilaments and β-III tubulin.^12^^,^^13^

No study so far explores the effect of combining dopamine and vitamin E in reprogramming MSCs toward a neuronal lineage. In this study, we aimed to investigate the effects of supplementing neuronal induction media with dopamine and vitamin in promoting the neuronal differentiation of MSCs. We hypothesize that combining dopamine and vitamin E can enhance the rate of MSC differentiation toward dopaminergic neurons, which can open up new avenues for improving MSC-based therapy for neurodegenerative diseases.

## METHODS

### Cell line

Human adipose tissue-derived MSCs (hAD-MSCs) was commercially purchased from Lonza Group (Basel, Switzerland) (Cat# PT5006, Lot# 21TL138912). MSCs were expanded using Dulbecco’s Modified Eagle’s Medium Low Glucose (DMEM-Low Glucose, Euroclone, Pero, Italy), which contained 5.6 mmol/L glucose and were supplemented with 10% fetal bovine serum (Gibco, Waltham, MA, United States),^14^ 0.1 mg/mL streptomycin, and 100 units/mL penicillin in standard cell culture incubator (5% CO_2_/95% air; 37°C). Medium was changed every 72 hours and cells were passaged when confluency was over 70%.

### Neural differentiation

The cryopreserved hAD-MSCs were thawed and seeded on fibronectin-coated 12-wells plates (Corning, Corning, NY, United States, Cat#354402) at a density of 2 × 10^3^ cells/cm^2^ in MSCs medium (Day 0) for 24 hours. The medium was then changed to neural induction medium (Cytiva, Marlborough, MA, USA, Cat #SH30781158) for 10 days with media change every second day. Other sets of study groups included induction of hAD-MSCs using the same neural induction medium with addition of dopamine and vitamin E. The first group had the addition of 2.5 µM dopamine and 12.5 µM vitamin E. The second group had the addition of 5 µM dopamine and 25 µM vitamin E. Neural differentiation was evaluated by immunocytochemistry and Western blotting.

### Reagents and antibodies

Antibodies used for Western blotting and immunostaining: Nestin (Santa Cruz Biotechnology, Dallas, TX, United States, Cat #sc-377380), TBR1 (Santa Cruz Biotechnology, Cat # sc-376258), SATB2 (Santa Cruz Biotechnology, Cat #sc-81376), MAP2 (Santa Cruz Biotechnology, Cat #sc-74421), choline acetyltransferase (CHAT) (GenoChem, Cat #GW0042R), dopamine transporter (DAT) (Santa Cruz Biotechnology, Cat #sc-32258), dopamine beta-hydroxylase (DBH) (Santa Cruz Biotechnology, Cat # sc-365710), NeuN (Genochem, Cat #GW1613R), NeuN (Santa Cruz Biotechnology, Cat #sc-33684), Phalloidin-iFluor 488 Reagent (Abcam, Cambridge, United Kingdom, Cat #ab176753), Anti-mouse Alexa Fluor 647 (Invitrogen, Waltham, MA, United States), Anti-rabbit Alexa Fluor 647 (Invitrogen, United States), Mounting Medium With DAPI (Abcam, United Kingdom), Ultra High Sensitivity ECL Kit (GlpBio, Montclair, CA, United States, Cat# GK10008).

### Immunocytochemistry

The plated cells were washed with phosphate buffered saline (PBS) and fixed with 4% paraformaldehyde. The fixed cells were permeabilized using 0.2% Triton X in PBS for 15 minutes at room temperature. The cells were then incubated with a blocking agent (2% bovine serum albumin in PBS for 20 minutes at room temperature) to prevent non-specific binding, followed by incubation with the respective primary and secondary fluorescent antibodies. The cells were counter-stained with mounting media containing DAPI (4′,6-diamidino-2-phenylindole) to visualize the nuclei. The slides were then imaged using a Cytation 5 imaging system (BioTek Instruments, Winooski, VT, United States).

### Western blotting

The protein levels of DAT, CHAT, and NeuN were measured by Western blotting. Briefly. The plated cells were scraped using cold PBS and pelleted. The cells pellet was re-suspended in protein lysis buffer (RIPA with protease inhibitors). Total protein levels were measured using NanoDrop Lite Spectrophotometer, and 40 μg of protein was loaded onto SDS-PAGE. Following electrophoresis, proteins were transferred to polyvinylidene fluoride (PVDF) membranes and were incubated with appropriate primary and secondary antibodies. The membranes were visualized using VILBER FUSION Gel Documentation System, and bands were quantified using ImageJ for densitometry and normalized to β-actin.

### Statistical analysis

Data are reported as mean  ±  SD. Comparison of data between multiple groups was performed using one-way analysis of variance (ANOVA) followed by Tukey’s post-hoc multiple comparison test, and analysis between 2 groups was made using the Student t-test (two-tailed). Statistical significance is determined as *P* <.05. Each figure represents one of at least 3 independent quantifiable experiments.

## RESULTS

### Morphological characteristics of the differentiated cells

**Figure 1.**
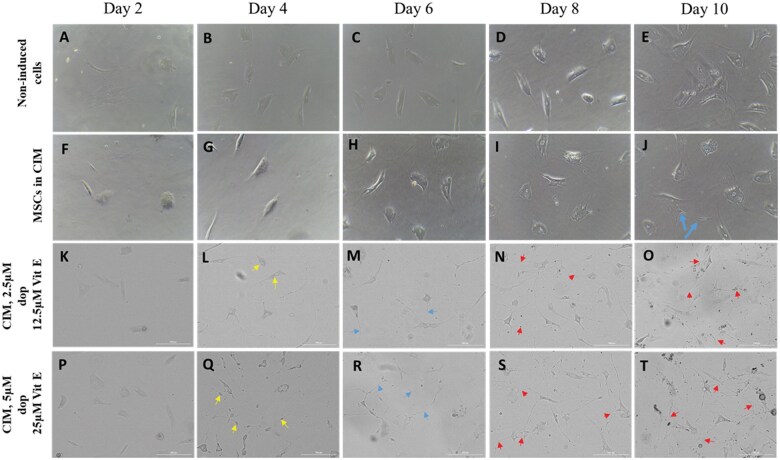
Neural differentiation of hMSCs. (A-E) MSCs cultured in non-inductive low-glucose media. After 10 days, no signs of differentiation were observed. (F-J) MSCs cultured in conventional induction media (CIM). By day 10, morphological changes were evident, with some cells exhibiting extensions arising from the cell body (blue arrows). (K-O) MSCs cultured in CIM supplemented with 2.5 µM dopamine (dop) and 12.5 µM vitamin E (Vit E). Morphological changes were noticeable after 48 hours, with cells becoming rounder (yellow arrows) and developing distinct dendrite-like and axon-like structures at day 6 (blue arrows), from day 8 to 10 the formation of synaptic connections between cells (red arrows). (P-T) MSCs cultured in IM supplemented with 5 µM dop and 25 µM Vit E. Morphological changes occurred more rapidly and were more pronounced compared to the lower-dose group, demonstrating enhanced differentiation. Original magnification: ×10.

During the induction period, morphological changes in control and induced hAD-MSCs were observed under an inverted microscope. The non-induced cells consistently retained their fibroblast-like morphology, characterized by a flattened, spindle-shaped appearance, abundant cytoplasm, and prominent nuclei, showing no signs of neuronal differentiation throughout the observation period (Figure 1A-E). In contrast, cells exposed to conventional induction media (CIM) began to exhibit neural-like morphologies by day 10, forming cell bodies (Figure 1F-J). Remarkably, cells treated with media enriched with dopamine and vitamin E underwent distinct morphological changes after 48 hours of neuronal induction. Initially, they displayed irregular shapes and sizes, which progressively transformed into rounded forms. This marked the onset of neuronal differentiation, as these rounded cells extended new neurites (Figure 1L and Q). Within 96 hours post-induction, these neurites matured, developing into well-defined dendrite-like and axon-like structures, indicative of advanced neuronal characteristics.

By day 6, the Dopa-Vit E-induced MSCs show significant progress in neuronal morphogenesis. The cells developed more pronounced and elongated neurite networks, resembling the precursors of axons and dendrites (Figure 1M and R). These neurites often extended in various directions, sometimes forming interconnected webs between neighboring cells. Growth cones, small, dynamic structures at the tips of these neurites, can be observed, indicating active cytoskeletal remodeling.

From days 8 to 10, the Dopa-Vit E-induced MSCs exhibit increasingly mature neuronal characteristics (Figure 1N, O, S, and T). The neurite networks become more refined, with distinct axons and dendrites clearly identifiable. Axons grow longer and exhibit branching patterns, while dendrites develop secondary and tertiary extensions, enhancing their complexity. The overall morphology closely resembles that of mature neurons’ and the cells display a polarized structure typical of functional neurons. One of the most significant observations during this stage was the formation of synaptic connections between cells. Synaptic vesicles and presynaptic terminals began to appear.

### Neuronal differentiation markers

**Figure 2.**
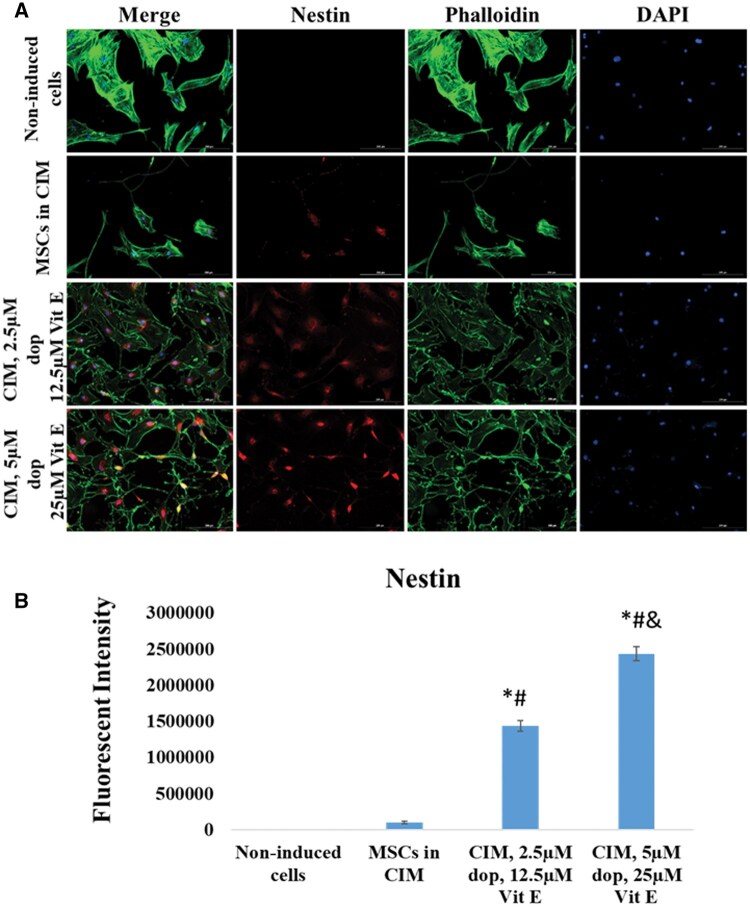
Nestin expression. (A) Representative images of Nestin expression in non-induced MSCs cells, in cells treated with conventional induction media (CIM), and in cells with CIM supplemented with 2 concentrations of dopamine (dop) and vitamin E (Vit E). (B) Fluorescence intensity of Nestin expression in the study groups. (*P <.05 versus non-induced control, # *P* < .05 versus MSCs in CIM, and *P* <.05 versus CIM with 2.5 µM dop and 12.5 µM Vit E (*n* = 3)).

**Figure 3.**
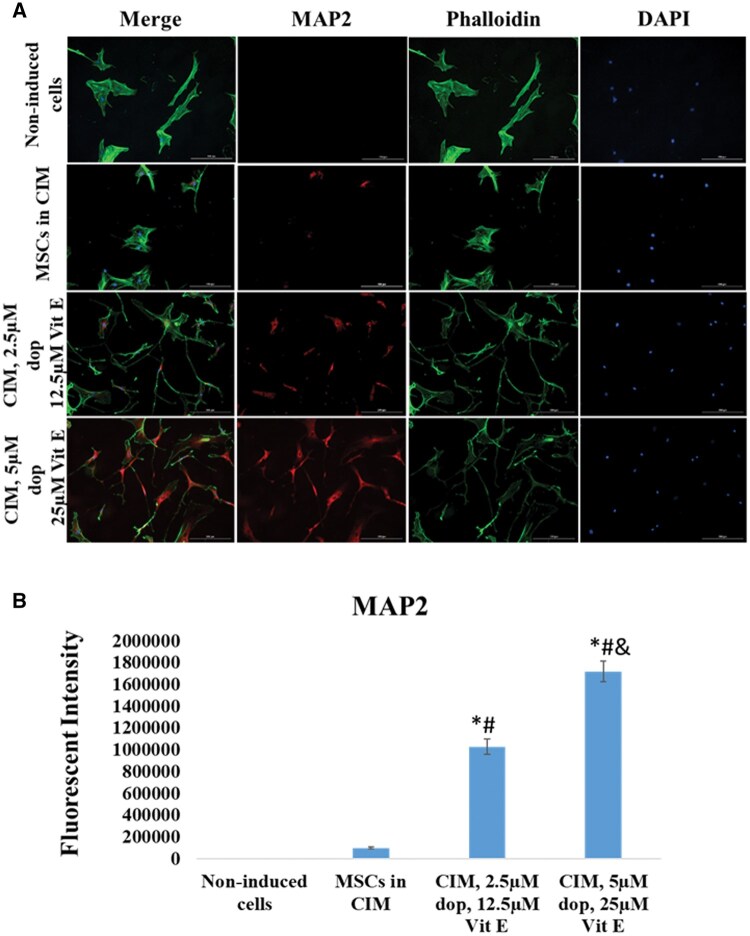
MAP2 expression. (A) Representative images of MAP2 expression in non-induced MSCs cells, in cells treated with conventional induction media (CIM), and in cells treated with CIM supplemented with 2 concentrations of dopamine (dop) and vitamin E (Vit E). (B) The fluorescence intensity of MAP2 expression in the study groups. (*P <.05 versus non- induced control, # *P* <.05 versus MSCs in CIM, and *P* <.05 versus CIM with 2.5 µM dop and 12.5 µM Vit E (*n* = 3)).

**Figure 4.**
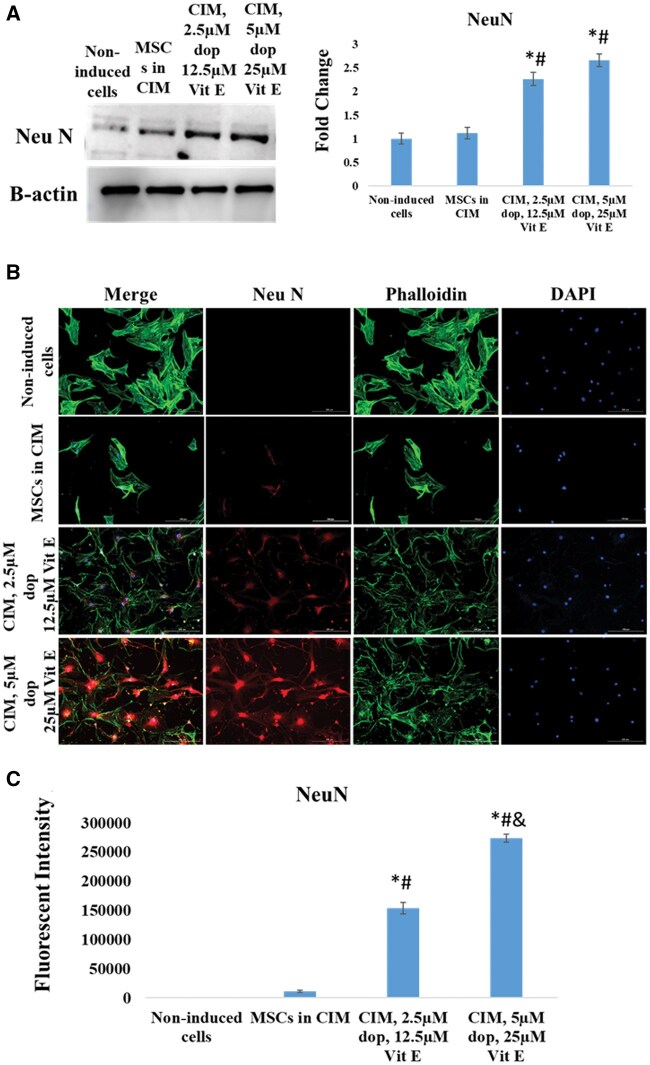
NeuN expression. (A) The expression of NeuN protein level in non-induced MSCs cells, cells treated with conventional induction media (CIM), cells treated with CIM supplemented with 2 concentrations of dopamine (dop) and vitamin E (Vit E). (B) Representative images of NeuN expression in the study groups. (C) Fluorescence intensity of NeuN expression in the study groups. (*P <.05 versus non-induced control, # *P* <.05 versus MSCs in CIM, and *P* <.05 versus CIM with 2.5 µM dop and 12.5 µM Vit E (*n* = 3)).

At the end of neural induction period, immunocytochemistry examination showed that both cells treated with CIM and cells treated with conventional media supplemented with dopamine and vitamin E expressed Nestin (a marker of neural stem cells and neural progenitor stem cells) (Figure 2) and MAP2 (marker of mature neurons) (Figure 3) compared to absent or low expression in non-treated MSCs. Similarly, immunocytochemistry and Western blotting examinations showed that the treated cells were positive for NeuN (marker of mature neurons) (Figure 4). Interestingly, the expression of these 3 markers was shown to be significantly higher in Dopa-Vit E-induced MSCs (in a dose-dependent manner) compared to cells treated with CIM, suggesting higher potential for neuronal differentiation of MSCs with addition of dopamine and vitamin E.

**Figure 5.**
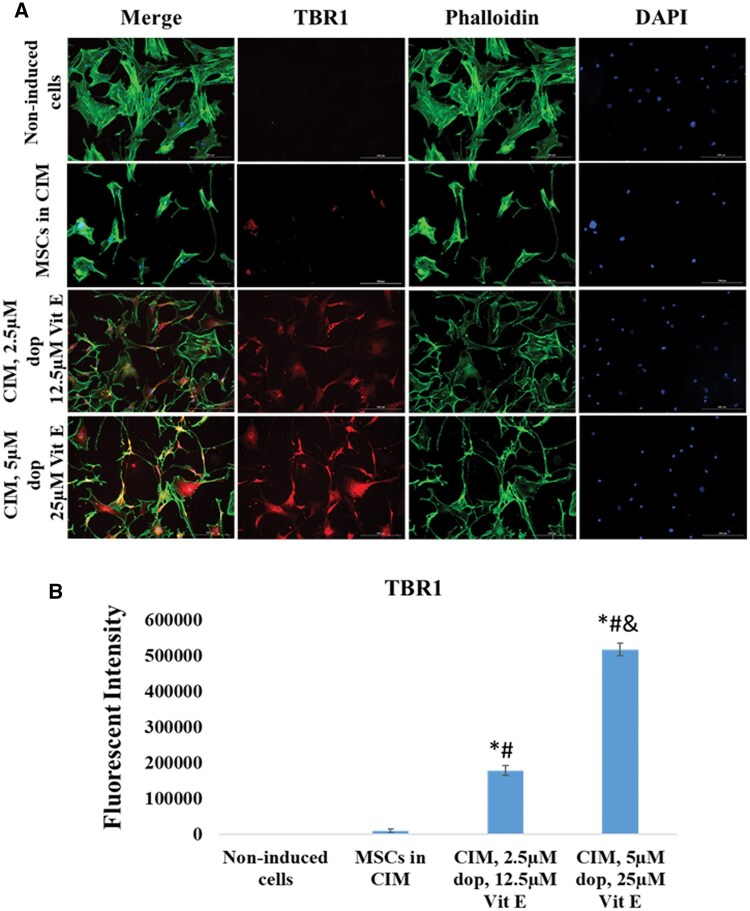
TBR1 expression. (A) Representative images of TBR1 expression in non-induced MSCs cells, cells treated with conventional induction media (CIM), and cells treated with CIM supplemented with 2 concentrations of dopamine (dop) and vitamin E (Vit E). (B) Fluorescence intensity of TBR1 expression in the study groups. (*P <.05 versus non-induced control, #*P* <.05 versus MSCs in CIM, and *P* <.05 versus CIM with 2.5 µM dop and 12.5 µM Vit E (*n* = 3)).

**Figure 6.**
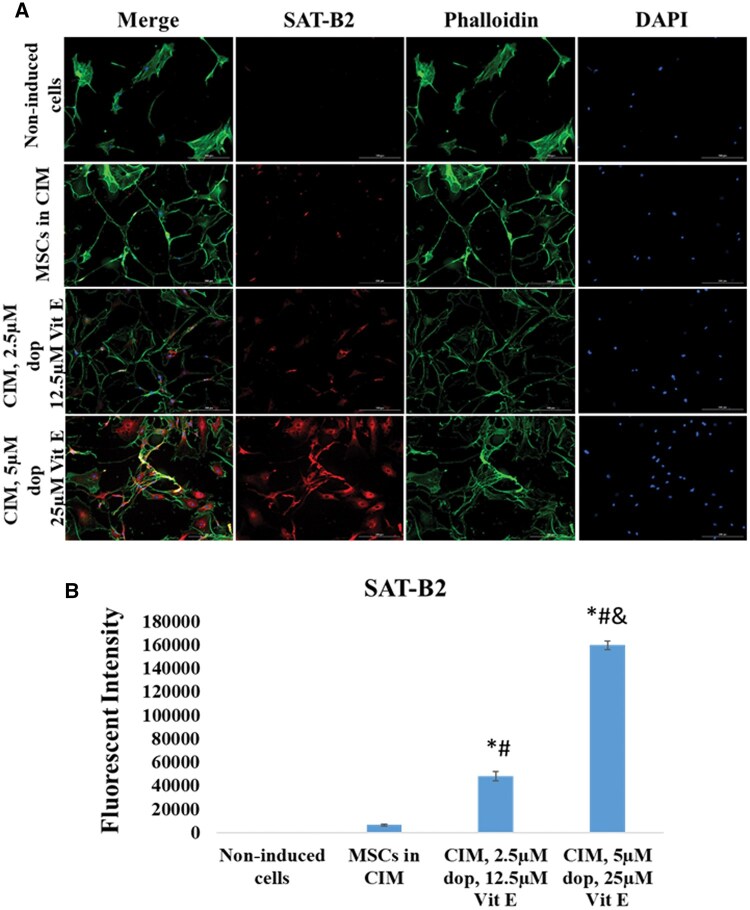
SAT-B2 expression. (A) Representative images of SAT-B2 expression in non-induced MSCs cells, in cells treated with conventional induction media (CIM), and cells treated with CIM supplemented with 2 concentrations of dopamine (dop) and vitamin E (Vit E). (B) Fluorescence intensity of SAT-B2 expression in the study groups. (*P <.05 versus non-induced control, #*P* <.05 versus MSCs in CIM, and *P* <.05 versus CIM with 2.5 µM dop and 12.5 µM Vit E (*n* = 3)).

To further examine the neuronal maturation in the cultured cells, we investigated the expression of TBR1 and SATB2. TBR1 is a marker of deep cortical layer neurons and its expression is essential for several critical early events in cortical development.^15^ SATB2 is a marker of superficial cortical layer neurons and plays a significant role in axon guidance and synaptic connectivity in the developing cortex.^16^ Therefore, the expression of these 2 markers in the differentiated cells suggests that they have committed to a mature neuronal state. Our results showed that both markers were significantly upregulated in Dopa-Vit E-induced MSCs compared to those cultured in CIM (Figures 5 and 6). These findings highlight the potent effects of dopamine and vitamin E in fostering the neuronal maturation of MSCs.

### The expression of dopaminergic and cholinergic neuronal proteins

**Figure 7.**
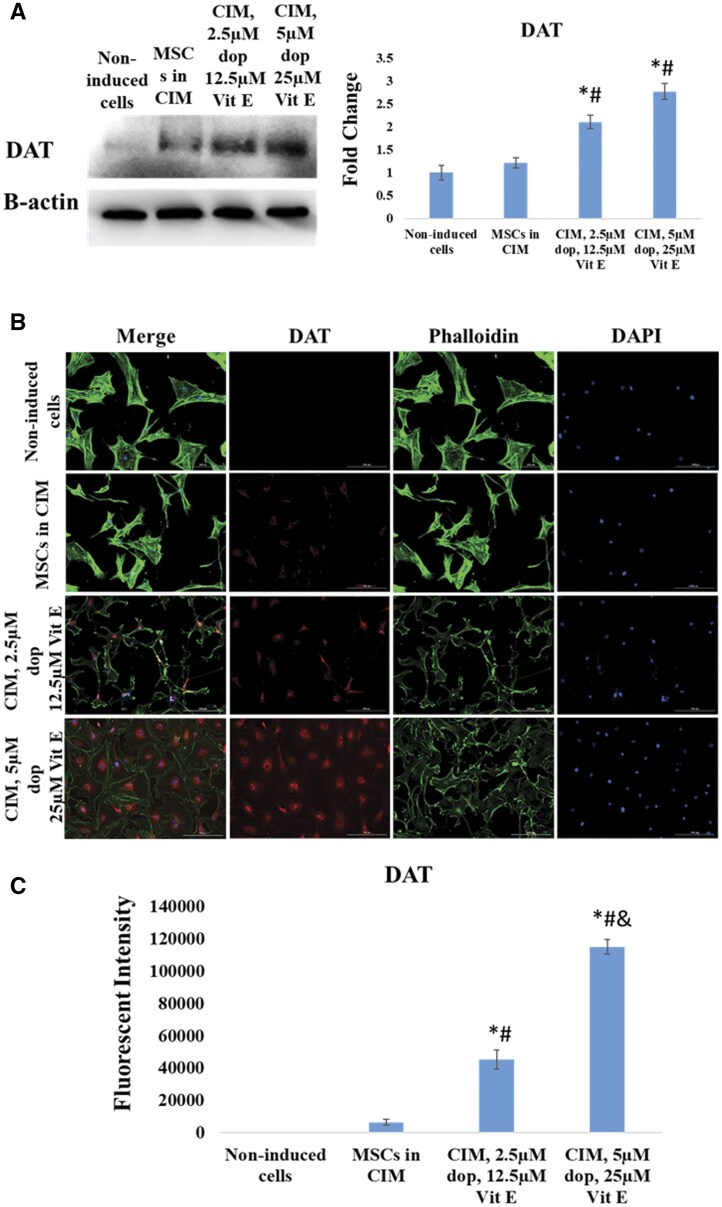
DAT expression. (A) DAT protein levels in non-induced MSCs cells, cells treated with conventional induction media (CIM), and in cells treated with CIM supplemented with 2 concentrations of dopamine (dop) and vitamin E (Vit E). (B) Representative images of DAT expression in the study groups. (C) Fluorescence intensity of DAT expression in the study groups. (*P <.05 versus non-induced control, #*P* <.05 versus MSCs in CIM, and *P* <.05 versus CIM with 2.5 µM dop and 12.5 µM Vit E (*n* = 3)).

**Figure 8.**
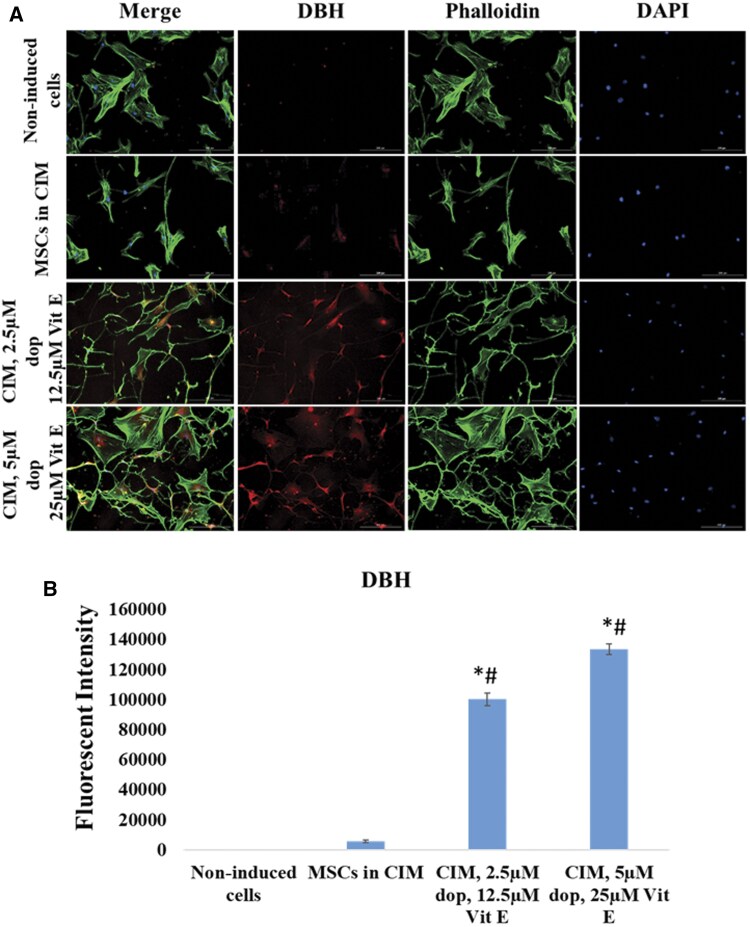
DBH expression. (A) Representative images of DBH expression in non-induced MSCs cells, cells treated with conventional induction media (CIM), and in cells treated with CIM supplemented with 2 concentrations of dopamine (dop) and vitamin E (Vit E). (B) Fluorescence intensity of DBH expression in the study groups. (*P <.05 versus non-induced control, #*P* <.05 versus MSCs in CIM (*n* = 3)).

**Figure 9.**
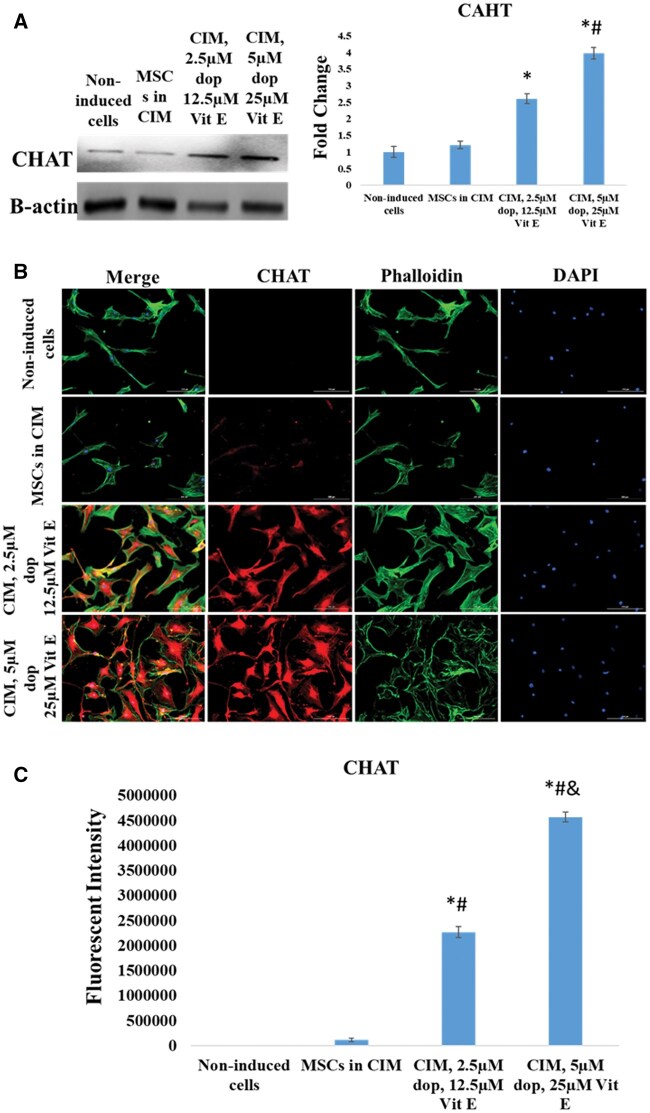
CHAT expression. (A) CHAT protein levels in non-induced MSCs cells, cells treated with conventional induction media (CIM), and in cells treated with CIM supplemented with 2 concentrations of dopamine (dop) and vitamin E (Vit E). (B) Representative images of CHAT expression in the study groups. (C) Fluorescence intensity of CHAT expression in the study groups. (*P <.05 versus non-induced control, #*P* <.05 versus MSCs in CIM, and *P* <.05 versus CIM with 2.5 µM dop and 12.5 µM Vit E (*n* = 3)).

Finally, we investigated the presence of different types of neurons (such as dopaminergic and cholinergic neurons) in the cell cultures after induction. We evaluated the expression of DAT and DBH, which are markers of dopaminergic neurons, and the expression of CHAT, an enzyme linked to cholinergic neurons. Our results showed that the expression of these 3 markers were greatly upregulated in Dopa-Vit E-induced MSCs compared to those cultured in CIM (Figures 7-9), indicating that addition of dopamine and vitamin E significantly enhanced differentiation toward these sub-specifications of neurons.

## DISCUSSION

Enhancing the differentiation of MSCs into neurons offers a promising alternative for treating neurodegenerative diseases. Reprogramming MSCs to specifically acquire neuronal characteristics is considered challenging and requires extensive investigation to find optimized reprogramming protocols that are efficient and ensure neuronal functionality. The neuronal differentiation of MSCs faces several challenges that limit their potential for therapeutic applications and regenerative medicine.^17^ One major issue is achieving efficient and consistent differentiation into functional, mature neurons.^3^ MSC-derived neuronal cells often exhibit limited complexity, such as underdeveloped dendritic processes and synaptic structures, and may lack full functional integration with existing neural networks.^3^ Another challenge lies in optimizing the microenvironment and induction protocols to mimic the complex signals present in native neuronal differentiation.^3^ Additionally, concerns about scalability, reproducibility, and maintaining the genetic and phenotypic stability of MSCs during the differentiation process remain significant hurdles.^3^ The potential for incomplete or aberrant differentiation further complicates their use in clinical settings. Overcoming these challenges requires a deeper understanding of the signaling pathways involved, as well as the development of innovative strategies to enhance the differentiation efficiency and functionality of MSC-derived neurons.

The current study aims to investigate the potential role of dopamine and vitamin E in optimizing the microenvironment to enhance the differentiation of MSCs into developed neurons. By integrating these factors into the induction protocols, the study seeks to address existing challenges in MSC neuronal differentiation, such as limited maturation and functionality. The ultimate goal is to develop a more robust and reproducible method for generating expendable, fully functional neurons with applications in regenerative medicine and neurological research.

This study highlights the remarkable potential of MSCs to progression toward neuronal lineage when exposed to dopamine and vitamin E. Within 2 days, MSCs transitioned into neural progenitor-like cells, marked by the expression of early neuronal commitment indicators. Over the 10 subsequent days, these progenitor cells underwent phenotypic maturation into fully differentiated neurons, characterized by the expression of markers indicative of neuronal identity and functionality.

The differentiation of MSCs into neurons involves the sequential expression of specific markers corresponding to different stages of neuronal maturation and development.^3^^,^^18^ In this study, neuronal markers were selected to comprehensively evaluate the neuronal differentiation of MSCs after being conditioned using dopamine and vitamin E at different stages of neuronal maturation and specification. Early-stage markers such as Nestin, which is an indicative marker of neural stem and progenitor cells,^19^ were used to identify initial differentiating commitment.

MAP2 serves as a critical marker of neuronal maturation, reflecting advanced stages of differentiation.^20^ Its significantly higher expression in Dopa-Vit E-induced MSCs underscores the positive effects of supplementing dopamine and vitamin E in driving MSCs toward neuronal lineages. MAP2 is associated with dendritic development, which signified mature neuronal morphology.^20^

CHAT, DAT, and DBH are key markers used to assess the differentiation of MSCs into specific neuronal subtypes.^3^^,^^21–23^ CHAT is an enzyme essential for the synthesis of acetylcholine, a neurotransmitter in cholinergic neurons,^24^^,^^25^ while DAT and DBH marked the formation of dopaminergic neurons.^26^^,^^27^ DBH, an enzyme responsible for converting dopamine to norepinephrine, creates a balanced neurogenic microenvironment.^28^ DAT also regulates the dopamine reuptake ensuring optimal dopamine signaling in dopaminergic neurons and proper expression of neuronal markers like βIII-tubulin and tyrosine hydroxylase.^29^^,^^30^

Furthermore, the post-mitotic neuronal markers TBR1^31^ and SATB2^32^^,^^33^ showed a significant increase in their expression in Dopa-Vit E-induced MSCs compared to those cultured in CIM or non-induced controls. The upregulation in these post-mitotic neuronal markers highlighted the formation of mature neurons which exited the cell cycle permanently and entered G0 phase.

NeuN is a nuclear protein predominantly expressed in post-mitotic neurons where it is involved in regulating gene expression essential for neuronal function and survival.^34^ The remarkable upregulation in the level of NeuN in Dopa-Vit E-induced MSCs also indicated that these cells have attained the typical characteristics of mature neurons.

Interestingly, the substantial increase in the level of CHAT, DAT, and DBH reflected the commitment of Dopa-Vit E-induced MSCs to cholinergic and dopaminergic lineages. Thus, the concomitant expression of these markers after supplementing the induction media with dopamine and vitamin E can enhance the neuronal differentiation ability of MSCs toward functionally distinct neuronal subtypes.

More specifically, our study shows that the expression level of CHAT was approximately 2-folds higher in Dopa-Vit E-induced MSCs compared to DAT and DBH. This differential expression of these markers suggested that Dopa-Vit E-induced MSCs were most likely reprogrammed toward cholinergic neuronal lineage. The presence of a small percentage of glutamatergic and dopaminergic neurons following the addition of dopamine and vitamin E highlighted the heterogeneity of the formed neuronal cells. The formation of heterogeneous neuronal cells can be attributed to the variation in cellular sensitivity and receptor expression profiles of MSC populations, ie, some cells may predominantly acquire dopaminergic differentiation ability while others may primarily react to vitamin E only and differentiate to glutamatergic neurons. Despite this heterogeneity, our study found that the majority of MSCs may respond synergistically to dopamine and vitamin E and develop features of cholinergic neurons, highlighting the complexity of MSCs neuronal reprogramming and the need to optimize the differentiation protocol. Future studies must focus in investigating the functional capabilities of these neurons in vivo and ensuring the regenerative ability of the reported induction protocol for degenerative neuronal diseases. The current study provides promising strategy for generating functional neurons and holds neoteric potential to improve the MSCs-based therapy for neurodegenerative diseases by replenishing the damaged neuronal cells.

## Data Availability

The data that supports the findings in this study are available from the corresponding authors upon reasonable request.

## References

[1] Gadhave DG , Sugandhi VV, Jha SK, et al Neurodegenerative disorders: mechanisms of degeneration and therapeutic approaches with their clinical relevance. Ageing Res Rev. 2024;99:102357. 38830548 10.1016/j.arr.2024.102357

[2] Kvistad CE , Kråkenes T, Gavasso S, et al Neural regeneration in the human central nervous system—from understanding the underlying mechanisms to developing treatments. Where do we stand today? Front Neurol. 2024;15:1398089. 38803647 10.3389/fneur.2024.1398089 PMC11129638

[3] Hernández R , Jiménez-Luna C, Perales-Adán J, et al Differentiation of human mesenchymal stem cells towards neuronal lineage: clinical trials in nervous system disorders. Biomol Ther (Seoul). 2020;28:34-44. 31649208 10.4062/biomolther.2019.065 PMC6939692

[4] Berebichez-Fridman R , Montero-Olvera PR. Sources and clinical applications of mesenchymal stem cells: state-of-the-art review. Sultan Qaboos Univ Med J. 2018;18:e264-e277. 30607265 10.18295/squmj.2018.18.03.002 PMC6307657

[5] Han Y , Yang J, Fang J, et al The secretion profile of mesenchymal stem cells and potential applications in treating human diseases. Signal Transduct Target Ther. 2022;7:247572027. 10.1038/s41392-022-00932-0 PMC8935608 35314676

[6] Mishra A , Singh S, Shukla S. Physiological and functional basis of dopamine receptors and their role in neurogenesis: possible implication for Parkinson’s disease. J Exp Neurosci. 2018;12:1179069518779829. 29899667 10.1177/1179069518779829 PMC5985548

[7] Corkrum M , Araque A. Astrocyte-neuron signaling in the mesolimbic dopamine system: the hidden stars of dopamine signaling. Neuropsychopharmacology. 2021;46:1864-1872. 34253855 10.1038/s41386-021-01090-7 PMC8429665

[8] Klein MO , Battagello DS, Cardoso AR, et al Dopamine: functions, signaling, and association with neurological diseases. Cell Mol Neurobiol. 2019;39:31-59. 30446950 10.1007/s10571-018-0632-3 PMC11469830

[9] Gaggi G , di Credico A, Izzicupo P, et al Human mesenchymal stromal cells unveil an unexpected differentiation potential toward the dopaminergic neuronal lineage. Int J Mol Sci. 2020;2:221635710. 10.3390/ijms21186589 PMC7555006 32916865

[10] Traber MG. Vitamin E: necessary nutrient for neural development and cognitive function. Proc Nutr Soc. 2021;80:319-326. 33896432 10.1017/S0029665121000914 PMC8842820

[11] Ghasemi F , Khoshmirsafa M, Safari E, et al Vitamin E and selenium improve mesenchymal stem cell conditioned media immunomodulatory effects. Stem Cell Investig. 2021;8:9. 10.21037/sci-2020-008 PMC8173293 34124232

[12] Yu Z , Wang LM, Yang WX. How vitamin E and its derivatives regulate tumour cells via the MAPK signalling pathway? Gene. 2022;808:145998. 34626718 10.1016/j.gene.2021.145998

[13] Yang P , Zhao J, Hou L, et al Vitamin E succinate induces apoptosis via the PI3K/AKT signaling pathways in EC109 esophageal cancer cells. Mol Med Rep. 2016;14:1531-1537. 27357907 10.3892/mmr.2016.5445 PMC4940098

[14] Khasawneh RR , Sharie AHA, Rub EAE, et al Addressing the impact of different fetal bovine serum percentages on mesenchymal stem cells biological performance. Mol Biol Rep. 2019;46:4437-4441. 31154604 10.1007/s11033-019-04898-1

[15] Darbandi SF , Schwartz SER, Qi Q, et al Neonatal TBR1 dosage controls cortical layer 6 connectivity. Neuron. 2018;100:831-845.e7. 30318412 10.1016/j.neuron.2018.09.027 PMC6250594

[16] Leone DP , Heavner WE, Ferenczi E, et al SATB2 regulates the differentiation of both callosal and subcerebral projection neurons in the developing cerebral cortex. Cereb Cortex. 2015;25:3406-3419. 25037921 10.1093/cercor/bhu156 PMC4585495

[17] Gavasso S , Kråkenes T, Olsen H, et al The therapeutic mechanisms of mesenchymal stem cells in MS—a review focusing on neuroprotective properties. Int J Mol Sci. 2024;25:267224909. 10.3390/ijms25031365 PMC10855165 38338644

[18] George S , Hamblin MR, Abrahamse H. Differentiation of mesenchymal stem cells to neuroglia: in the context of cell signalling. Stem Cell Rev Rep. 2019;15:814-826. 31515658 10.1007/s12015-019-09917-z PMC6925073

[19] Bernal A , Arranz L. Nestin-expressing progenitor cells: function, identity and therapeutic implications. Cell Mol Life Sci CMLS. 2018;75:2177-2195. 29541793 10.1007/s00018-018-2794-z PMC5948302

[20] DeGiosio RA , Grubisha MJ, MacDonald ML, et al More than a marker: potential pathogenic functions of MAP2. Front Mol Neurosci. 2022;15:974890. 36187353 10.3389/fnmol.2022.974890 PMC9525131

[21] Almalki SG , Agrawal DK. Key transcription factors in the differentiation of mesenchymal stem cells. Differentiation. 2016;92:41-51. 27012163 10.1016/j.diff.2016.02.005 PMC5010472

[22] MacDonald HJ , Kleppe R, Szigetvari PD, et al The dopamine hypothesis for ADHD: an evaluation of evidence accumulated from human studies and animal models. Front Psychiatry. 2024;15:1492126. 39619336 10.3389/fpsyt.2024.1492126 PMC11604610

[23] Tunbridge EM , Narajos M, Harrison CH, et al Which dopamine polymorphisms are functional? systematic review and meta-analysis of COMT, DAT, DBH, DDC, DRD1–5, MAOA, MAOB, TH, VMAT1, and VMAT2. Biol Psychiatry. 2019;86:608-620. 31303260 10.1016/j.biopsych.2019.05.014

[24] Oda Y. Choline acetyltransferase: the structure, distribution and pathologic changes in the central nervous system. Pathol Int. 1999;49:921-937. 10594838 10.1046/j.1440-1827.1999.00977.x

[25] Wurtman RJ , Cansev ME, Ulus IH. Choline and its products acetylcholine and phosphatidylcholine. In: Lajtha A, Tettamanti G, Goracci G, eds. *Handbook of Neurochemistry and Molecular Neurobiology*. Springer; 2009:443-501. 10.1007/978-0-387-30378-9_18

[26] Nepal B , Das S, Reith MEA, et al Overview of the structure and function of the dopamine transporter and its protein interactions. Front Physiol. 2023;14:1150355. 36935752 10.3389/fphys.2023.1150355 PMC10020207

[27] Björklund A , Dunnett SB. Dopamine neuron systems in the brain: an update. Trends Neurosci. 2007;30:194-202. 17408759 10.1016/j.tins.2007.03.006

[28] Rahman N , Buck J, Levin L. pH sensing via bicarbonate-regulated “soluble” adenylyl cyclase (sAC). Front Physiol. 2013;4:343. 24324443 10.3389/fphys.2013.00343 PMC3838963

[29] Vaughan RA , Foster JD. Mechanisms of dopamine transporter regulation in normal and disease states. Trends Pharmacol Sci. 2013;34:489-496. 23968642 10.1016/j.tips.2013.07.005 PMC3831354

[30] Mulvihill KG. Presynaptic regulation of dopamine release: role of the DAT and VMAT2 transporters. Neurochem Int. 2019;122:94-105. 30465801 10.1016/j.neuint.2018.11.004

[31] Bedogni F , Hodge RD, Elsen GE, et al TBR1 regulates regional and laminar identity of postmitotic neurons in developing neocortex. Proc Natl Acad Sci. 2010;107:13129-13134. 20615956 10.1073/pnas.1002285107 PMC2919950

[32] Britanova O , Romero C de J, Cheung A, et al SATB2 is a postmitotic determinant for upper-layer neuron specification in the neocortex. Neuron. 2008;57:378-392. 18255031 10.1016/j.neuron.2007.12.028

[33] Turovsky EA , Turovskaya MV, Fedotova EI, et al Role of SATB1 and SATB2 transcription factors in the glutamate receptors expression and Ca2+ signaling in the cortical neurons in vitro. Int J Mol Sci. 2021;22:235299668. 10.3390/ijms22115968 PMC8198236 34073140

[34] Vv G , De K. NeuN as a neuronal nuclear antigen and neuron differentiation marker. Acta Naturae. 2015;7:42-47. 26085943 PMC4463411

